# Differential Roles of Orexin Receptors in the Regulation of Sleep/Wakefulness

**DOI:** 10.3389/fendo.2013.00057

**Published:** 2013-05-16

**Authors:** Michihiro Mieda, Natsuko Tsujino, Takeshi Sakurai

**Affiliations:** ^1^Department of Molecular Neuroscience and Integrative Physiology, Graduate School of Medical Science, Kanazawa UniversityKanazawa, Ishikawa, Japan

**Keywords:** orexin, hypothalamus, sleep, monoamine, narcolepsy, arousal

## Abstract

Orexin A and orexin B are hypothalamic neuropeptides that play critical roles in the regulation of sleep/wakefulness, as well as in a variety of physiological functions such as emotion, reward, and energy homeostasis. The actions of orexins are mediated by two receptors, orexin 1 (OX1R) and orexin 2 (OX2R) receptors. OX1R and OX2R show partly overlapping but distinct distributions throughout the central nervous system, suggesting their differential roles. This review presents and discusses the current knowledge concerning the physiological roles of each orexin receptor subtype, focusing on the regulation of sleep/wakefulness.

## Introduction

The neuropeptides orexin A and orexin B (also known as hypocretin 1 and hypocretin 2), were originally identified as endogenous ligands for two orphan G-protein-coupled receptors (Sakurai et al., 1998). Both orexin A and orexin B are derived from a common precursor peptide, prepro-orexin. An mRNA encoding the same precursor peptide of hypocretin 1 (corresponding to orexin A) and hypocretin 2 (orexin B) was independently identified by de Lecea et al. (1998) as a hypothalamus-specific transcript.

The actions of orexins are mediated by two receptors, orexin 1 (OX1R) and orexin 2 (OX2R) receptors (also known as HCRTR1 and HCRTR2) (Sakurai et al., 1998). OX1R shows a higher affinity for orexin A than orexin B by one-order, while OX2R binds orexin A and orexin B with similar affinities. Both receptors are coupled to G_q/11_ subclass of G proteins and caused strong excitatory effects on neurons examined thus far (Sakurai and Mieda, 2011). OX2R has also been reported to couple to G_i/o_ in a neuronal cell line when overexpressed (Zhu et al., 2003).

Orexins are produced in a specific population of neurons (orexin neurons) that are located exclusively in the hypothalamus, including the lateral hypothalamic area (LHA), perifornical area, and posterior hypothalamus (PH) (de Lecea et al., 1998; Peyron et al., 1998; Sakurai et al., 1998; Date et al., 1999; Nambu et al., 1999). Orexin neurons project in almost all brain areas with especially dense projections to monoaminergic neurons, which play important roles in the regulation of sleep/wake states. Orexins are thought to be a critical regulator of sleep/wake states. Intracerebroventricular (ICV) administration of orexin A and orexin B increases wakefulness and suppresses both rapid-eye-movement (REM) sleep and non-REM (NREM) sleep (Hagan et al., 1999). More importantly, orexin deficiency causes sleep disorder narcolepsy in humans and animals (Chemelli et al., 1999; Lin et al., 1999; Peyron et al., 2000; Thannickal et al., 2000; Hara et al., 2001). At present, various reports suggest that orexins are involved not only in the sleep/wake regulation but also in other physiological and behavioral processes such as food intake, emotion, stress response, and reward via activation of OX1R and OX2R (Yamanaka et al., 2003; Akiyama et al., 2004; Mieda et al., 2004; Boutrel et al., 2005; Harris et al., 2005; Sakurai et al., 2005; Narita et al., 2006; Yoshida et al., 2006). Recent reports revealed that each orexin receptor has a different function in these processes. Thus, understanding such a difference is important for understanding the function of the orexin system.

This review will discuss the physiological roles of the orexin system in the regulation of sleep/wakefulness, especially focusing on the functions of each orexin receptor.

## Distributions of Orexin Receptor mRNA

According to data obtained by *in situ* hybridization histochemistry, many brain regions have been shown to differentially express *OX1R* and *OX2R* mRNAs (Trivedi et al., 1998; Lu et al., 2000; Greco and Shiromani, 2001; Marcus et al., 2001). Consistent with the broad projections of orexin neurons, *OX1R* and *OX2R* show wide distributions within the brain with partly overlapping but distinct and complementary distributions. For example, *OX2R* is expressed in layers 2 and 6 throughout the cerebral cortex, while *OX1R* is expressed in layers 5 and 6, except the cingulate cortex where *OX1R* mRNA is found in layer 3. In the ammon’s horn of the hippocampus, CA1 and CA2 express *OX1R*, while CA3 expresses *OX2R*.

In the arcuate nucleus and paraventricular nucleus of the hypothalamus, which are involved in the regulation of feeding, energy homeostasis, autonomic and endocrine systems, *OX2R* is almost exclusively observed. Concerning the nuclei implicated in sleep/wake regulation, the locus ceruleus (LC), laterodorsal tegmental nucleus (LDT), and pedunculopontine tegmental nucleus (PPT) mainly express *OX1R* mRNA, while the tuberomammillary nucleus (TMN) almost exclusively expresses *OX2R* mRNA. The dorsal raphe (DR) and median raphe (MnR) express both subtypes. These distributions suggest partly overlapping and partly distinct roles of these two receptors.

**Figure 1:**
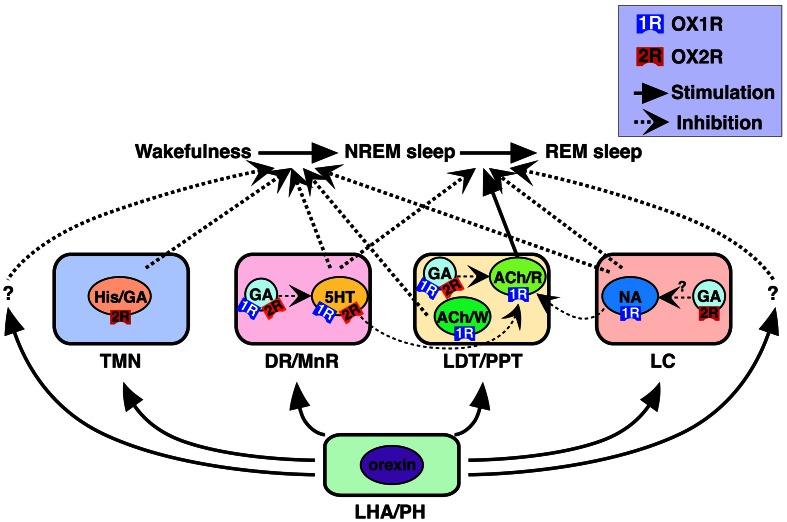
**Schematic illustration of presumed pathways underlying orexin actions on NREM and REM sleep (Mieda et al., 2011)**. Orexins activate histaminergic (His)/GABAergic (GA), serotonergic (5HT), noradrenergic (NA), and cholinergic (ACh) neurons as well as GABAergic putative interneurons in wake-promoting nuclei, including the TMN, DR/MnR, LDT/PPT, and LC. These neurons differentially express OX1R and/or OX2R, and regulate wakefulness/NREM sleep and NREM/REM sleep transitions. OX1R and OX2R may be expressed in the same populations of GABAergic neurons, as shown in the figure, or may be expressed in distinct populations of these neurons in each area. Wake/REM-on cholinergic neurons (ACh/W) are likely to suppress NREM sleep, but REM-on cholinergic neurons (ACh/R), are likely to induce REM sleep. Wake-active serotonergic and noradrenergic neurons in the DR/MnR and LC, respectively, counteract activation of REM-on cholinergic neurons in the LDT/PPT, as well as REM-on neurons in the brainstem reticular formation (Pace-Schott and Hobson, 2002; Sakurai, 2007). Previous reports have suggested contributions of GABAergic interneurons inhibiting PPT cholinergic and raphe serotonergic neurons (Liu et al., 2002; Takakusaki et al., 2005). LHA, lateral hypothalamic area; PH, posterior hypothalamus. Reproduced from Sakurai and Mieda (2011) with permission.

Monoaminergic (i.e., histaminergic, noradrenergic, and serotonergic) and cholinergic neurons in these nuclei have been considered critical for sleep/wakefulness regulation (Pace-Schott and Hobson, 2002; Zeitzer et al., 2006). Our recent work determined precise cellular localization of two orexin receptors in these nuclei (Figure 1) (Mieda et al., 2011). All histaminergic neurons in the TMN exclusively expressed *OX2R*, while all noradrenergic neurons in the LC exclusively expressed *OX1R*. In the DR and MnR, approximately 90% of serotonergic neurons expressed *OX1R* and/or *OX2R*. In addition, many non-serotonergic cells in the DR/MnR also expressed *OX1R* or *OX2R* mRNA. At least some populations of these cells were likely to be GABAergic, since a population of *Gad1*-positive cells also expressed detectable *OX1R* or *OX2R* mRNA. In the LDT and PPT, all cholinergic neurons expressed *OX1R* but not *OX2R* mRNA, while many *OX1R*-positive and/or *OX2R*-positive non-cholinergic neurons were intermingled with cholinergic neurons in the area. *Gad1* mRNA staining further revealed that *OX1R*- or *OX2R*-expressing cells included both GABAergic and non-GABAergic cells.

Orexin receptors-positive GABAergic neurons in the DR/MnR and LDT/PPT, which are intermingled with serotonergic and cholinergic neurons, respectively, are intriguing. Since GABAergic neurons are likely to function as local inhibitory interneurons and orexin receptors usually cause excitatory effects on neurons, simultaneous activation of serotonergic/cholinergic neurons with GABAergic interneurons by orexins within a nucleus may inhibit serotonergic/cholinergic neurons. Thus, balance of orexinergic activation between principal neurons and interneurons may be of importance. This point will be further discussed in a later section.

## Orexin and Monoaminergic/Cholinergic Systems

Orexin neurons project almost all brain regions with especially dense projections being seen in monoaminergic and cholinergic nuclei involved in the regulation of sleep/wakefulness. Various reports suggested that orexin excites these neurons directly and/or indirectly *in vivo* and *in vitro* (Figure 1). This orexinergic regulation may be important to control sleep/wakefulness. Here, we will review *in vitro* and *in vivo* electrophysiological studies of orexin receptors in monoaminergic and cholinergic neurons.

### Serotonergic neurons of the dorsal raphe

In 2001, Brown et al. reported that orexin A strongly excited DR serotonergic neurons in brain slices of rats under the whole-cell patch clamp recording. The depolarization persisted in the presence of the voltage dependent Na^+^ channel blocker, tetrodotoxin, indicating a direct postsynaptic action on the serotonin cells (Brown et al., 2001). Moreover, orexin A and orexin B induced inward currents with similar amplitude and dose-dependency in most serotonergic neurons (Brown et al., 2001; Liu et al., 2002; Soffin et al., 2004) via activation of Na^+^/K^+^ non-selective cation channels (Liu et al., 2002). These results may suggest that OX2R is mainly involved in the activation of serotonergic neurons, although single-cell PCR, *in situ* hybridization, and immunohistochemical studies revealed that serotonergic neurons express both *OX1R* and *OX2R* (Brown et al., 2001; Liu et al., 2002; Wang et al., 2005; Mieda et al., 2011).

A recent report revealed that orexin A depolarized DR neurons by activating a noisy inward current (Kohlmeier et al., 2004, 2008). This current was through non-selective cation channels that did not contribute to the somatic Ca^2+^ influx. These reports suggested that orexin A has two independent actions: activation of non-selective cation channels and activation of a protein kinase C-dependent enhancement of Ca^2+^ transients mediated by L-type Ca^2+^ channels.

At higher concentrations, orexin also increased spontaneous inhibitory postsynaptic currents in serotonergic neurons indicating orexin excited GABAergic interneurons that project to serotonergic neurons in the DR (Liu et al., 2002). Such a structure of intra-DR circuit may execute a negative feedforward regulation of the activation of serotonergic neurons by orexin neurons.

Regulation of DR neurons by orexin seems to be even more complex. Haj-Dahmane and Shen found that orexin B depresses the evoked glutamate-mediated synaptic currents in DR serotonergic neurons. This effect was mediated by retrograde endocannabinoid release, which depended on the stimulation of postsynaptic orexin receptors and subsequent activation of phospholipase C and DAG lipase enzymatic pathways but not on a rise in postsynaptic intracellular calcium (Haj-Dahmane and Shen, 2005).

*In vivo* extracellular recording also revealed that application of orexin A increased firing frequency of serotonergic DR neurons in unanesthetized rats during REM sleep or slow-wave sleep, while during wakefulness, a similar amount of orexin A did not increase the firing rate (Takahashi et al., 2005). These reports suggest that orexin excited DR serotonergic neurons in a behavioral state-dependent manner.

### Noradrenergic system

Orexin A and orexin B increased the firing frequency of all LC neurons in the presence of tetrodotoxin in rat brain slices by intracellular recording (Horvath et al., 1999; Soffin et al., 2002). The effects of orexin A are fivefold more potent than orexin B. Moreover, the effect was inhibited by selective orexin 1 receptor antagonist, SB-334867 (Soffin et al., 2002, 2004). These data suggest that orexins directly excited LC noradrenergic neurons via OX1R, which is consistent with exclusive expression of *OX1R* in these neurons demonstrated by *in situ* hybridization (Mieda et al., 2011). The orexin-induced depolarization of LC noradrenergic neurons may be produced by augmentation of the non-selective cationic conductance and suppression of G-protein-coupled inward rectifier (GIRK) channel activity (Hoang et al., 2003; Murai and Akaike, 2005).

In addition to the action of orexins within the LC, orexin A and B also evoked noradrenaline release in the rat cerebrocortical slice, suggesting that orexins act on OX1R on the orexin fibers projecting from the LC in the cerebral cortex (Hirota et al., 2001). Orexin-evoked noradrenaline release was time- and concentration-dependent and partially extracellular Ca^2+^-dependent (Hirota et al., 2001).

Another report suggested that the orexin modulates LC functions via OX1R by regulating noradrenaline release not only from the axonal terminals, but also from the somatodendritic region of LC noradrenergic neurons (Chen et al., 2008). Application of orexin alone dose-dependently induced somatodendritic noradrenaline release. Orexin A also potentiated *N*-methyl-d-aspartate (NMDA) receptor-mediated somatodendritic noradrenaline release from LC neurons, which was blocked by SB-334867, an OX1R antagonist, or a PKC inhibitor, indicating the involvement of OX1R and PKC. Orexin A enhanced NMDA-induced intracellular Ca^2+^ elevation as well (Chen et al., 2008). Taken together, activation of OX1R of LC noradrenergic neurons regulates not only noradrenergic input to their targets, but also noradrenergic communication in the soma and dendrites.

Excitatory effects of orexin A on LC neurons have been demonstrated also *in vivo* by measuring their firing rates (Bourgin et al., 2000) or noradrenaline release into the hippocampus (Walling et al., 2004). In addition, local administration of orexin A but not orexin B in the LC of rats suppressed REM sleep in a dose-dependent manner and increased wakefulness at the expense of REM sleep and deep slow-wave sleep (Bourgin et al., 2000).

### Histaminergic system

Both patch clamp study using freshly isolated neurons and intracellular recording using brain slices revealed that orexin A and orexin B directly and dose-dependently depolarized TMN histaminergic neurons of rats (Bayer et al., 2001; Eriksson et al., 2001; Yamanaka et al., 2002). Orexin A and B showed almost the same potency, indicating that orexin-induced excitation of histaminergic neurons is mediated via OX2R. Consistent with electrophysiological data, *OX2R* was shown to be the principle subtype of orexin receptors by single-cell RT-PCR study, although both receptor mRNAs were detected in most tuberomammillary neurons (Eriksson et al., 2001). Histological studies have demonstrated that TMN histaminergic neurons express *OX2R* almost exclusively (Yamanaka et al., 2002; Mieda et al., 2011).

Orexin-induced depolarization was associated with a small decrease in input resistance. The mechanisms of depolarization of histaminergic neurons are likely to be related to the activation of both the electrogenic Na^+^/Ca^2+^ exchanger and Ca^2+^ current (Eriksson et al., 2001), as well as suppression of GIRK channels (Hoang et al., 2003).

On the other hand, a recent *in vitro* optogenetic study demonstrated that postsynaptic currents and rapid increase of firing rates in TMN histaminergic neurons evoked by stimulation of orexin neurons were mediated by glutamate rather than by orexins (orexin neurons are also glutamatergic) (Schone et al., 2012). This suggests a possibility that orexin might play as a modulator of the glutamatergic transmission in these cells.

*In vivo* application of orexin A to the TMN increased histamine release in both the medial preoptic area and the frontal cortex in a dose-dependent manner (Huang et al., 2001). Moreover, perfusion of orexin A into the TMN of rats through a microdialysis probe promptly increased wakefulness, concomitant with a reduction in REM and NREM sleep (Huang et al., 2001; Yamanaka et al., 2002). These findings indicate that orexins excite TMN histaminergic neurons mainly via OX2R and enhance histamine release from the TMN to maintain wakefulness.

### Cholinergic system

#### Laterodorsal tegmental nucleus

Whole-cell recordings revealed that orexin exhibited actions both presynaptically and postsynaptically on LDT cholinergic neurons, as well as in non-cholinergic neurons in the same area. Orexin increased frequency and amplitude of spontaneous EPSCs in these neurons. Postsynaptically, orexin produced an inward current and an increase in membrane current noise, which were accompanied by a conductance increase (Burlet et al., 2002). Being similar to DR serotonergic neurons, orexin A has two independent actions on LDT cholinergic neurons: activation of non-selective cation channels and activation of a protein kinase C-dependent enhancement of Ca^2+^ transients mediated by L-type Ca^2+^ channels (Kohlmeier et al., 2004, 2008).

In a study of *in vivo* extracellular recordings of LDT neurons in rats, the application of orexin A induced long-lasting excitation in five of seven cholinergic neurons (Takahashi et al., 2002). Furthermore, when orexin A was microinjected in the LDT of cats, time spent in wakefulness was significantly increased due to an increase in the duration of wake episodes, and time spent in REM sleep was significantly reduced due to a decrease in the frequency of active sleep episodes (Xi et al., 2001).

#### Pedunculopontine tegmental nucleus

Whole-cell patch clamp recordings revealed that orexin A and orexin B depolarized PPT cholinergic and non-cholinergic neurons dose-dependently in the presence of tetrodotoxin (Kim et al., 2009a). Approximately 80% of PPT neurons were depolarized by orexin A, and 20% of PPT neurons did not respond to orexin A (Kim et al., 2009b). SB-334867, a selective inhibitor for OX1R, significantly suppressed the orexin A-induced depolarization. These results suggest that orexin depolarized PPT neurons directly through OX1R. This OX1R-mediated depolarization was caused by a decrease of K^+^ conductance and an increase of non-selective cationic conductance (Kim et al., 2009a). It was also indicated that it was blocked by D609, a phosphatidylcholine-specific PLC inhibitor, suggesting that the excitatory effects of orexin on PPT neurons are mediated by PLC-dependent pathway (Kim et al., 2009b).

An *in vivo* study demonstrates that orexin inhibited PPT neurons via GABAergic neurons (Takakusaki et al., 2005). In decerebrated cats, an injection of orexin into the PPT increased the intensity of electrical stimulation required at the PPT to induce muscle atonia. The effect of orexin on PPT was abolished by simultaneous injection of bicuculline, a GABA_A_ receptor antagonist, into the PPT. These results suggest that orexin enhances GABAergic effects on presumably cholinergic PPT neurons *in vivo* to control postural muscle tone.

Cholinergic neurons in these areas include two functionally distinct types of neurons. Some are active during wakefulness and REM sleep (W/REM-on neurons) and others are specifically active during REM sleep (REM-on neurons) (Pace-Schott and Hobson, 2002; Sakurai, 2007). The latter population is likely to play a critical role in REM sleep-related physiological phenomena, including muscle atonia. Orexin A excites both cholinergic and non-cholinergic neurons of the LDT in slice preparations, as described previously (Burlet et al., 2002). Furthermore, orexin A microinjection in the cat LDT increases wakefulness and reduces REM sleep. In addition, as mentioned previously, GABAergic neurons in the PPT mediate suppression of REM sleep and muscle atonia following local injection of orexin A into this area (Takakusaki et al., 2005). Consistent with the actions of orexins on both cholinergic and non-cholinergic neurons, orexin receptors are differentially expressed in cholinergic and GABAergic neurons of the LDT/PPT, shown by a histological study (Mieda et al., 2011). Taken together, these results indicate that in the LDT/PPT, orexin may activate W/REM-on cholinergic neurons through OX1R to facilitate wakefulness. Simultaneously, orexin might activate GABAergic interneurons to inhibit REM-on cholinergic neurons in these nuclei (Figure 1).

#### Basal forebrain

Electrophysiological studies were performed on cultured nucleus basalis neurons from the basal forebrain and on rat brain slice (Eggermann et al., 2001; Hoang et al., 2004). In cultured neurons, orexin A induced a depolarization and increased firing accompanied by a decrease of whole-cell conductance in cholinergic neurons. The mechanism of this neuronal excitation was shown to be accompanied by inhibition of inward rectifier K^+^ channel (KirNB) activity (Hoang et al., 2004). In rat brain slices, orexin has a direct excitatory effect on the cholinergic neurons of the contiguous basal forebrain (Eggermann et al., 2001). All cholinergic neurons were excited by orexin A and even more potently by orexin B. This result suggests that the action depends on OX2R. Thus, orexins may excite cholinergic neurons in the basal forebrain via OX2R and contribute to the cortical activation.

The medial septum-diagonal band of Broca (MSDB), via its cholinergic and GABAergic projections to the hippocampus, controls the hippocampal theta rhythm and associated learning and memory functions. MSDB receives a dense innervation of orexin neurons, and neurons of the MSDB express very high levels of OX2R. Septohippocampal cholinergic neurons were excited by orexin A and B with similar EC_50_ in a concentration-dependent manner, mediated via a direct postsynaptic mechanism (Wu et al., 2004). The orexin effect is likely to be mediated by inhibition of a K^+^ current, presumably an inward rectifier, and activation of the Na^+^–Ca^2+^ exchanger. Thus orexin effects within the septum should increase hippocampal acetylcholine release and thereby promote hippocampal arousal (Wu et al., 2004).

## Differential Roles of Each Orexin Receptor Subtype in the Regulation of Sleep/Wakefulness

### Molecular genetic studies

Recent studies have established that the orexin (hypocretin) system is one of the most important regulators of sleep/wake states and that its deficiency results in the human sleep disorder narcolepsy (Zeitzer et al., 2006; Sakurai and Mieda, 2011). Narcolepsy is characterized by excessive daytime sleepiness that often results in “sleep attacks” (sudden onset of NREM sleep), cataplexy (sudden bilateral skeletal muscle weakening triggered by emotions without loss of consciousness), hypnagogic hallucinations, and sleep paralysis. These symptoms can be divided into two independent pathological phenomena. One is the inability to maintain a consolidated awake period characterized by abrupt transitions from wakefulness to NREM sleep (i.e., dysregulation of NREM sleep onset). This phenomenon manifests clinically as excessive daytime sleepiness or sleep attacks. The other key phenomenon is the pathological intrusion of REM sleep into wakefulness or at sleep onset (i.e., dysregulation of REM sleep onset). It is during these periods that patients experience cataplexy, hypnagogic hallucinations, and sleep paralysis.

Mice with targeted deletion of the *prepro-orexin* gene (*orexin^−/−^* mice) display a phenotype strikingly similar to narcolepsy: abrupt behavioral arrests with muscle atonia (cataplexy), fragmented wakefulness (inability to maintain consolidated wakefulness episodes), and direct transitions from wakefulness to REM sleep (Chemelli et al., 1999). In addition, functionally null mutations in the *OX2R* gene were found in two independent lines of familial narcoleptic dogs (Lin et al., 1999).

Based on studies on orexin receptor-deficient mice (*OX1R^−/−^* and *OX2R^−/−^* mice), it has been suggested that deletion of *OX1R* produces no measurable effect on sleep/wakefulness states (Sakurai, 2007; Hondo et al., 2010). However, *OX2R^−/−^* mice have clear characteristics of narcolepsy, although their behavioral and EEG phenotype is less severe than that found in *orexin^−/−^* mice (Willie et al., 2003). In infrared videophotographic studies in the dark phase, *OX2R^−/−^* mice showed abrupt behavioral arrests. However, its frequency was much less than in *orexin^−/−^* mice (31-fold lower frequency in *OX2R^−/−^* mice as compared to *orexin^−/−^* mice). Instead, *OX2R^−/−^* mice showed a distinct variety of behavioral arrests with onsets that were more gradual in nature (gradual arrests). Moreover, *orexin^−/−^* mice also exhibit gradual arrests with a frequency similar to *OX2R^−/−^* mice in addition to plenty of abrupt arrests.

In contrast to abrupt arrests, gradual arrests typically began during quiet wakefulness and could be easily distinguished from the normal onset of resting behavior by (i) the absence of stereotypic preparation for sleep (e.g., nesting and/or assumption of a curled or hunched posture with limbs drawn under the body) and (ii) a characteristic ratchet-like “nodding” of the head over a period of several seconds with a transition to a collapsed posture (Willie et al., 2003).

Detailed observations of behaviors during EEG/EMG recordings found that abrupt arrests in *orexin^−/−^* and *OX2R^−/−^* mice occurred during direct transitions from wakefulness to REM sleep, whereas EEG/EMG correlates of gradual arrests in both *orexin^−/−^* and *OX2R^−/−^* mice invariably revealed the onset of attenuated muscle tone, but not atonia, and an EEG transition from wakefulness to NREM sleep (Chemelli et al., 1999; Willie et al., 2003). Pharmacologically, abrupt arrests in *orexin^−/−^* mice were suppressed by systemic administration of clomipramine, an anticataplectic agent used for treatment of human narcolepsy. Whereas, administration of caffeine, a psychostimulant used to treat excessive sleepiness in human narcolepsy, tended to produce a mild exacerbation of abrupt arrest frequency. In clear contrast, systemic administration of caffeine dose-dependently suppressed gradual arrests, while administration of an anticataplectic agent clomipramine did not affect the frequency of gradual arrests in both *orexin^−/−^* and *OX2R^−/−^* mice. These observations showed strong similarity with those observed in human narcolepsy patients; psychostimulant drugs are effective for the sleepiness, but exacerbate cataplexy, which is treatable with antidepressants. This detailed characterization of behavioral, pharmacological, and electrophysiological features of *orexin^−/−^* and *OX2R^−/−^* mice defined abrupt and gradual arrests as the presumptive mouse correlates of cataplexy and sleep attack in human narcolepsy, respectively.

In addition to the gradual behavioral arrests, *OX2R^−/−^* mice exhibit fragmentation of wakefulness, another sign of sleepiness, to the extent similar to that in *orexin^−/−^* mice (Willie et al., 2003). *OX1R^−/−^*; *OX2R^−/−^* mice appear to have the same phenotype with *orexin^−/−^* mice, implying that these two receptors are sufficient to mediate regulation of sleep/wakefulness by orexins (Sakurai, 2007; Hondo et al., 2010). These results of mouse reverse genetic studies suggest that normal regulation of wake/NREM sleep transitions depends critically on OX2R activation, whereas the profound dysregulation of REM sleep control are unique to narcolepsy from loss of signaling through both OX1R- and OX2R-dependent pathways.

Substantially lower frequency of cataplexy in *OX2R^−/−^* mice as compared to *orexin^−/−^* mice appears to be inconsistent with the fact that mutations of *OX2R* gene are solely responsible for an inherited canine model of narcolepsy, which demonstrate frequent occurrence of cataplexy as well as excessive sleepiness (Lin et al., 1999). This may result from species difference (e.g., the precise expression patterns of two orexin receptors). However, even in dogs, the absence of orexin peptides may cause severe narcoleptic symptoms as compared to *OX2R* mutation. Early studies of narcoleptic Dobermans and Labradors found these dogs to be 30- to 80-fold less severely affected with cataplexy than poodles with sporadic narcolepsy that showed literally hundreds of attacks a day (Baker et al., 1982), an effect previously attributed solely to differences in breed and breed size.

As an experiment complementary to the behavioral studies and baseline sleep/wakefulness recordings of *OX1R^−/−^* and *OX2R^−/−^* mice, we elucidated that two orexin receptors play distinct and differential roles in the regulation of sleep and wakefulness states by comparing the effects of ICV orexin A administration in wild-type, *OX1R^−/−^* and *OX2R^−/−^* mice (Mieda et al., 2011). The effects of orexin A on wakefulness and NREM sleep were significantly attenuated in both knockout mice as compared to wild-type mice, with substantially larger attenuation in *OX2R^−/−^* mice than in *OX1R^−/−^* mice. These results suggest that although the OX2R-mediated pathway has a pivotal role in the promotion of wakefulness, OX1R also plays additional roles in promoting arousal.

In contrast, suppression of REM sleep by orexin A administration was slightly and similarly attenuated in both *OX1R^−/−^* and *OX2R^−/−^* mice, suggesting a comparable contribution of the two receptors to REM sleep suppression (Mieda et al., 2011). In addition, our observations further suggest that orexin A directly suppresses transitions from NREM sleep to REM sleep, and that activation of OX1R is sufficient for this effect. Supplementary role of OX1R in the suppression of NREM sleep is consistent with the fact that *OX2R^−/−^* mice on a C57BL/6J genetic background show less fragmented wakefulness when compared to *orexin^−/−^* mice and *OX1R^−/−^*; *OX2R^−/−^* mice (but show similarly fragmented wakefulness on a C57BL/6J-129/SvEv mixed background) (Sakurai, 2007; Mochizuki et al., 2011), suggesting that OX1R is indispensable for the maintenance of wakefulness in the absence of OX2R.

Although application of exogenous orexins has been shown to excite many types of neurons (Sakurai and Mieda, 2011), neurons directly downstream to orexin neurons in physiological conditions (i.e., neurons influenced by endogenous orexins that mediate their wake-promoting and REM-suppressing effects), have remained uncertain. Several reports suggested that histaminergic neurons in the TMN play an important role in the arousal-promoting effect of orexin, supported by the facts that the effect of ICV orexin A administration is markedly attenuated by the histamine H1 receptor antagonist pyrilamine (Yamanaka et al., 2002) and is absent in *H1 histamine receptor* knockout mice (Huang et al., 2001) and that the TMN expresses OX2R abundantly (Marcus et al., 2001; Mieda et al., 2011), the subtype whose absence causes narcoleptic phenotype in mice and dogs (Lin et al., 1999; Willie et al., 2003). Mochizuki et al. (2011) produced a mouse model in which a *loxP*-flanked gene cassette disrupts production of the OX2R, but normal OX2R expression can be restored by Cre recombinase. They showed that targeted Cre expression (i.e., focal restoration of OX2R expression), in the TMN and adjacent regions rescues fragmentation of wakefulness in this mouse model, suggesting that the orexin signaling mediated by OX2R in the TMN (and possibly its surrounding area in the PH) is sufficient to prevent sleepiness caused by systemic OX2R deficiency.

On the other hand, this hypothesis is still controversial. Mice lacking both OX1R and histamine H1 receptors demonstrate no abnormality in sleep/wakefulness (Hondo et al., 2010). In addition, a recent optogenetic study showed that orexin-mediated sleep-to-wake transitions do not depend on the TMN histaminergic neurons (Carter et al., 2009). Rather, another optogenetic study suggested the role of LC noradrenergic neurons as a direct downstream pathway of orexin neurons by demonstrating that optogenetic inhibition of LC noradrenergic neurons blocked arousal effects of optogenetic stimulation of orexin neurons (Carter et al., 2012).

### Pharmacological studies

Antagonists for orexin receptors are drawing people’s attention as novel medications for insomnia (Scammell and Winrow, 2011; Mieda and Sakurai, 2013). Several non-selective (dual) antagonists for orexin receptors, as well as subtype-selective antagonists, have been developed. These drugs are indeed useful also for studying the roles of each subtype in the regulation of sleep/wakefulness without considering any chronic compensatory changes that might complicate the phenotypes of genetically engineered models.

A study reported that an OX2R-selective antagonist JNJ10397049 has a better ability to promote NREM sleep than the dual antagonist almorexant in rats (Dugovic et al., 2009). They suggested that simultaneous inhibition of OX1R attenuates the sleep-promoting effects mediated by selective OX2R blockade, possibly correlated with dopaminergic neurotransmission. In contrast, another recent study led to a different conclusion (Morairty et al., 2012). It reported that an OX1R-selective antagonist SB-334867 produces small increases in REM and NREM sleep, and an OX2R-selective antagonist EMPA produces a significant increase in NREM sleep. But administration of almorexant increases NREM sleep more than these subtype-selective antagonists, leading to the conclusion that dual orexin receptor antagonism is more effective for sleep promotion than subtype-selective antagonism. The difference in these drugs’ capabilities of crossing the blood-brain barrier, as well as their pharmacokinetic/pharmacodynamic characters, might influence the effectiveness. The latter view is also consistent with the conclusion derived from studies using subtype-specific knockout mice that OX2R plays a pivotal role, but OX1R has additional effects on promotion of wakefulness (Sakurai, 2007; Mieda et al., 2011).

## Conclusion

The orexin system is a necessary component for the normal regulation of sleep/wakefulness. Nevertheless, broad expression of orexin receptors throughout the brain makes it difficult to identify neurons and orexin receptor subtypes that are directly regulated by endogenous orexins and mediate their effects on the physiology of interest in a natural context. Future studies using molecular genetic strategies such as brain region/cell type-specific deletions and brain region/cell type-specific rescues of orexin receptors, as well as pharmacological studies of focal administration of subtype-specific orexin agonists/antagonists, would further dissect the differential roles of orexin receptors in the regulation of sleep/wakefulness. Furthermore, the orexin system is also important for the regulation of a variety of physiological functions, such as feeding, reward, and emotions. Thus, precise information of the different roles of the two orexin receptors is beneficial not only for understanding the mechanisms underlying orexinergic regulations of physiology, but also for application of orexin agonists/antagonists as medications for various diseases.

## Conflict of Interest Statement

The authors declare that the research was conducted in the absence of any commercial or financial relationships that could be construed as a potential conflict of interest.
